# Circular RNA hsa_circRNA_102209 promotes the growth and metastasis of colorectal cancer through miR‐761‐mediated Ras and Rab interactor 1 signaling

**DOI:** 10.1002/cam4.3332

**Published:** 2020-07-24

**Authors:** Chi Li, Hong Zhou

**Affiliations:** ^1^ Department of General Surgery the First Affiliated Hospital of Jinzhou Medical University Jinzhou P.R. China; ^2^ Radiology the First Affiliated Hospital of Jinzhou Medical University Jinzhou P.R. China

**Keywords:** colorectal cancer, growth, hsa_circRNA_102209, metastasis, RIN1 signaling

## Abstract

In our study, has_circRNA_102209 was the most elevated regulator in colorectal cancer (CRC) tissues according to circRNA array data. The levels of hsa_circRNA_102209 in CRC specimens and cells, as well as its effects on CRC cells were investigated. The expression of hsa_circRNA_102209 in CRC and paired non‐cancerous samples, human CRC, and normal colonic epithelial cells were examined using reverse transcription‐quantitative polymerase chain reaction (RT‐qPCR). Cells with hsa_circRNA_102209 knockdown were established using lentiviral vectors. Cell proliferative ability was evaluated using CCK‐8 assay; cell migrative/invasive activities were determined using wound healing/Transwell assay. Cell cycle arrest and apoptosis were assessed by flow cytometry; apoptosis, and EMT markers were examined using RT‐qPCR and western blotting. Tumor development and levels of associated proteins were determined in hsa_circRNA_102209 knockdown mice. Our results revealed that expression of hsa_circRNA_102209 was remarkably increased in CRC tissues, where the levels of miR‐761 were notably reduced (*P* < .05). Additionally, the levels of hsa_circRNA_102209 were associated with histology grade and occurrence of liver metastasis in CRC patients, and the expression of hsa_circRNA_102209 and miR‐761 were negatively correlated (*P* < .05). Moreover, hsa_circRNA_102209 was upregulated in CRC cells compared with normal colonic epithelial cells. Knockdown of hsa_circRNA_102209 notably inhibited the proliferation, migration, invasion, and EMT of CRC cells (*P* < .05), whereas cell cycle arrest at G0/G1 phase and apoptosis were enhanced (*P* < .05). Furthermore, miR‐761/Ras and Rab interactor 1 (RIN1) axis was the putative target of hsa_circRNA_102209 in CRC and involved in hsa_circRNA_102209‐modulated growth and metastasis of CRC cells (*P *< .05). Knockdown of hsa_circRNA_102209 also remarkably suppressed tumor growth in vivo (*P* < .05). In summary, our data revealed that the expression of hsa_circRNA_102209 was elevated in CRC samples and cells. Furthermore, hsa_circRNA_102209 could promote the progression of CRC through miR‐761/RIN1 axis. More importantly, hsa_circRNA_102209/miR‐761/RIN1 signaling may be a novel therapeutic target for the treatment of CRC patients.

## Graphical abstract

Our data revealed that the expression of hsa_circ_102209 was elevated in CRC samples and cells. Furthermore, hsa_circ_102209 could promote the progression of CRC through miR‐761/RIN1 axis. More importantly, hsa_circ_102209/miR‐761/RIN1 signaling may be a novel therapeutic target for the treatment of CRC patients.

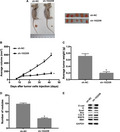

## 1 INTRODUCTION

Colorectal cancer (CRC) is one of the major causes of cancer‐associated mortality, and ~1.4 million new cases are diagnosed globally each year.^1^ CRC is a multifactorial disease and the pathogenesis is complex. Previous reports have indicated that both genetic and epigenetic alterations are associated with the initiation and development of CRC.^2^, ^3^, ^4^ Modern therapeutic strategies have been developed in recent years,^5^, ^6^ for instance, endoscopic and surgical resection are widely used for patients with CRC. Nevertheless, the prognosis of this disease is still poor due to high occurrence of liver metastases (~50%), which is one of the main causes of CRC‐related mortality.^7^, ^8^ The therapeutic outcome of patients with CRC is associated with disease staging when he/she is diagnosed, and 5‐year survival rate for CRC patients with metastases remains poor (<10%).^9^ Therefore, the molecular mechanisms underlying the development of CRC need to be further investigated, and it is essential to discover promising diagnostic biomarkers for this disease.

Circular RNAs (circRNAs) are a new class of noncoding RNAs. Unlike their linear counterparts, circRNAs are characterized with a more stable structure by forming a continuous loop.^10^, ^11^ As there are no free 5′‐/3′‐overhangs in circRNAs, they are resistant to exonuclease‐mediated cleavage.^12^ In addition, circRNAs are specifically expressed in various tissues.^13^ Recently, some circRNAs have been revealed as novel gene regulators, but the potential roles of most circRNAs remain largely unknown and require further investigation.^13^ Previous study has suggested that circRNAs may function as miRNA “sponges” which competitively suppress the activity of miRNAs.^14^ Furthermore, circRNAs are also involved in the pathogenesis of numerous types of diseases, such as nervous system disorders and cancer.^15^, ^16^, ^17^ Although a few circRNAs are novel gene regulators, the detailed functions of most circRNAs remain unknown.^10^ Furthermore, recent research has also elucidated the importance of circRNA‐miRNA‐mRNA regulatory network during tumorigenesis.^11^, ^12^ Recently, novel circRNAs have been identified as key factors in CRC pathogenesis. For instance, previous report has revealed the essential roles of a novel circRNA hsa_circ_0007534 in CRC, and it could promote the growth of cancer cells^18^; however, the underlying mechanisms and putative downstream molecules of most circRNAs in CRC have not been completely elucidated.

MicroRNAs (miRNAs) are noncoding RNAs with the length of ~22 nt, and they are potential downstream targets of other noncoding RNAs including lncRNAs and circRNAs.^19^, ^20^ Previous reports have suggested that miRNAs exert their functions via binding to the 3′ untranslated region of corresponding mRNAs.^21^, ^22^ Aberrant expression profiles of miRNAs have been revealed in patients with cancer, which can result in tumor development.^23^ For example, miR‐93 induces the proliferation of glioma cells through phosphatidylinositol 3 kinase/protein kinase B signaling.^22^ Upregulated H19 could enhance cell proliferation via miR‐675 in CRC.^20^ In addition, miR‐140 and miR‐152 interact with corresponding lncRNAs, consequently leading to the progression of glioma.^24^, ^25^ However, the potential functions of miRNAs in CRC remain elusive and require further investigation. Among the abovementioned miRNAs, miR‐761 is involved in the pathogenesis of gastric cancer by suppressing Ras and Rab interactor 1 (RIN1; 26).

In this study, the effects of hsa_circRNA_102209‐regulated signaling on the growth and metastasis of CRC cells were elucidated. Our data suggested that hsa_circRNA_102209 was upregulated in CRC tissues and cells. Knockdown of hsa_circRNA_102209 could inhibit the progression of CRC cells in vitro. In addition, miR‐761/RIN1 axis was the putative target of hsa_circRNA_102209 in CRC and involved in hsa_circRNA_102209‐modulated growth and metastasis of CRC cells. Knockdown of hsa_circRNA_102209 also remarkably suppressed tumor growth in vivo. In summary, our paper revealed the essential roles of hsa_circRNA_102209/miR‐761/RIN1 signaling during the development of CRC, which could provide novel insight for the treatment of this disease.

## 2 MATERIALS AND METHODS

### 2.1 CircRNA microarray

Total RNA was extracted using TRIzol reagent (Invitrogen; Thermo Fisher Scientific, Inc.) and RNeasy Mini Kit (Qiagen). Fluorescence‐labelled targets were generated for circRNA array. Human circRNA array v2 (CapitalBio Technology) was designed, ~5000 circRNAs were mounted onto the chip, target sequences of these circRNAs were obtained from Rybak‐Wolf 2015 and Circbase. Briefly, labeled targets were hybridized with the samples, which were scanned using Agilent Microarray Scanner (Agilent Technologies). Data were normalized according to Quantile algorithm. Arrays were carried out using the protocol provided by Agilent Technologies Inc. Shanghai Corporation. CircRNAs whose fold change >2 were further analyzed. Five most up‐ or downregulated circRNAs in CRC patients were listed in Table 1. Hsa_circRNA_102209 was selected for further study.

**Table 1:** Top five up‐/downregulated circRNAs in the array

CircRNA ID	Fold change	*P*‐value	circRNA type	Genomic location
Upregulated				
hsa_circ_0045890	4.92	.00672	Exonic	chr17
hsa_circ_0000735	3.56	.00721	Exonic	chr17
hsa_circ_0023642	2.92	.01123	Exonic	chr11
hsa_circ_0068610	2.13	.03015	Exonic	chr3
hsa_circ_0032821	2.09	.04234	Exonic	chr14
Downregulated				
hsa_circ_0005730	3.17	.04005	Exonic	chr5
hsa_circ_0003645	3.02	.02124	Exonic	chr16
hsa_circ_0026134	2.86	.01034	Exonic	chr12
hsa_circ_0061274	2.32	.01903	Exonic	chr21
hsa_circ_0092368	2.11	.00706	Exonic	chr1

### 2.2 Clinical specimens

Fifty‐six CRC and matched para‐carcinoma tissues (≥5 cm from tumor margin; aged 40‐78 years old; 26 males and 30 females) were obtained at the First Affiliated Hospital of Jinzhou Medical University (Jinzhou, China) during May 2010‐March 2013. After surgery, the specimens were immediately frozen using liquid nitrogen and stored at −80°C until further use. Prior to operation, none of the patients have received chemo‐ or radio‐therapy. Moreover no malignancy was found in other organs. The levels of hsa_circRNA_102209 were categorized into low/high group using the mean value. The biopsies were examined by two independent pathologists, and the clinicopathological features of enrolled patients were summarized in Table 2. Overall survival rates were analyzed using Kaplan‐Meier method. Written informed consents were obtained from patients, and all the samples were kept anonymized. The experimental protocol was approved by the Medical Ethics Committee of the First Affiliated Hospital of Jinzhou Medical University.

**Table 2:** Clinicopathological parameters of CRC patients enrolled in this study

Parameters	n	Hsa_circRNA_102209 expression		*P* value
		Low	High	
Gender				
Male	26	13	13	.515
Female	30	15	15	
Age (y)				
>60	24	12	12	.418
≤60	32	16	16	
Tumor size (cm)				
>5	24	13	11	.404
≤5	32	15	17	
Histology grade				
I‐II	34	22	12	.019^a^
III‐IV	22	6	16	
Smoking				
Yes	31	15	16	.433
No	25	13	12	
Liver metastasis				
Yes	22	5	17	.012^a^
No	34	23	11	

Note

Differences among variable were analyzed using the χ^2^ test.

^a^ The values have statistically significant differences.

### 2.3 Cell culture

One normal human colonic epithelial cell line (NCM460) as well as four human CRC cell lines (SW48, P6C, HT‐29 and Gp2d) were obtained from the American Type Culture Collection (Manassas, VA, USA). Cells were cultured using Dulbecco's modified Eagle's medium (DMEM) supplemented with streptomycin (100 µg/mL), penicillin (100 U/mL) and 10% fetal bovine serum (FBS; HyClone; GE Healthcare Life Science). Cells were cultured at 37°C in a humid incubator supplemented with 5% CO_2_.

### 2.4 Transfection

To generate hsa_circRNA_102209 or RIN1 overexpression model, wildtype (WT; o/e‐102209 or o/e‐RIN1) as well as mutant (o/e‐NC) fragment was inserted into PLCDH‐cir vector (Ribobio). In hsa_circRNA_102209 knockdown model, shRNA sequences against hsa_circRNA_102209 (sh‐102209) or negative control (sh‐NC) were obtained from Genepharm Co. Ltd. (Shanghai, China). After annealing, shRNA were integrated into lentiviral pU6‐Luc‐Puro vector (Genepharm Co. Ltd.). The lentiviral vectors were obtained from Hanbio (Shanghai, China). Transfection was carried out according to the manufacturer's protocols. CRC cells were selected by 0.5ug/mL puromycin (Sigma‐Aldrich) 2 weeks post‐transfection. To establish the knockdown model of hsa_circRNA_102209, pooled siRNA targeting hsa_circRNA_102209 (si‐102209) and negative control (si‐NC) were purchased from Genepharm Co. Ltd. The mimics/inhibitors of miR‐761 and corresponding negative control (NC) were synthesized by Genepharm Co. Ltd (Shanghai, China). The mimics/inhibitors (100pM) and siRNA (50 nM) were transfected into CRC cells using Lipofectamine^®^2000 (Invitrogen; Thermo Fisher Scientific, Inc.) according to the manufacturer's protocols. The culture medium was replenished with fresh DMEM supplemented with 10% FBS 8 hours post‐transfection. The transfection efficiency was evaluated using RT‐qPCR.

### 2.5 Reverse transcription‐quantitative polymerase chain reaction

Reverse transcription‐quantitative polymerase chain reaction (RT‐qPCR) was used to examine the expression levels of hsa_circRNA_102209, miR‐761 and RIN1 in each experimental group. MiRNA was isolated using miRNeasy Mini Kit (Qiagen). TaqMan MicroRNA Assay (Applied Biosystems) was used to determine the levels of miR‐761, and qPCR was performed on Applied Biosystem 7500. Total RNA from clinical samples or cell lines was extracted using TRIzol^®^ reagent (Invitrogen; Thermo Fisher Scientific, Inc.) according to the manufacturer's protocols. The concentration of eluted RNA was determined by NanoDrop 1000 spectrophotometer (Thermo Fisher Scientific, Inc.). Quality of isolated RNA was evaluated using Agilent 2100 Bioanalyzer (Agilent), and 28S:18S ratio was >1.0. Subsequently, cDNA was synthesized using a PrimeScript™ RT kit (Takara Biotechnology Co., Ltd), and qPCR was carried out using SYBR Green PCR Master Mix (TaKaRa Biotechnology Co., Ltd.) according to the manufacturer's protocols. Endogenous GAPDH and U6 were used as controls to normalize the expression of mRNA and miRNA. The sequences of forward and reverse primer used in the experiment were as follows: hsa_circRNA_102209, 5′‐GTGCAGAGAACTACATGTCACC‐3′ and 5′‐TAAAGGGTTGTCGCAGTGTG‐3′; miR‐761, 5′‐ACAGCAGGCACAGAC‐3′ and 5′‐GAGCAGGCTGGAGAA‐3′; RIN1, 5′‐GCACCTGGCGAGAGAAAAG‐3′ and 5′‐TAGATTTCCGCACGAGGA ACG‐3′; E‐cad, 5′‐GTATTTTAGTTTGGGTGAAAGAGTGAG‐3′ and 5′‐AAATACCTACAACAACAACAACAAC‐3′; Vimentin, 5′‐TCTACGAGGAGGAGATGCGG‐3′ and 5′‐GGTCAAGACGTGCCAGAGAC‐3′; Snail, 5′‐ACCACTATGCCGCGCTCTT‐3′ and 5′‐GGTCGTAGGGCTGCTGGAA‐3′; Bax, 5′‐GCCCTTTTGCTTCAGGGTTT‐3′ and 5′‐TCCAATGTCCAGCCCATGAT‐3′; Cas‐9, 5′‐GTTTGAGGACCTTCGACCAGCT‐3′ and 5′‐CAACGTACCAGGAGCCACTCTT‐3′; MMP9, 5′‐GGTGGACCGGATGTTCCC‐3′ and 5′‐GCCCACCTCCACTCCTCC‐3′; GAPDH, 5′‐GCAAGAGCACAAGAGGAAGA‐3′ and 5′‐ACTGTGAGGAGGGGAGATTC‐3′ and U6, 5′′‐CTCGCTTCGGCAGCACATA‐3′ and 5′AACGATTCACGAATTTGCGT‐3′. For thermocycler, PCR program used was as follows: 95°C for 5 minutes, then 45 cycles of 95°C for 15 seconds, 60°C for 20 seconds and 72°C for 10 seconds. Relative expression was analyzed using 2^‐∆∆Cq^ method.

### 2.6 Northern blotting

Total RNA was extracted using TRIzol^®^. Equal amount of RNA (30 μg) was added onto 15% TBE‐urea gels and separated. Then samples were transferred onto positively charged nylon membranes (GE Healthcare Life Sciences) and cross‐linked with UV irradiation. The blots were hybridized with DIG‐labelled probe for has_circRNA_102209 or miR‐761 (Exiqon) at 42°C overnight. Later on, the membranes were rinsed using low‐stringency buffer (2 × SSC containing 0.1% SDS). Then the levels of RNAs were evaluated using a DIG Luminescent Detection Kit (Roche). B‐actin or U6 was used as loading control, respectively.

### 2.7 Western blot analysis

Total protein from clinical samples or cells was extracted using radioimmunoprecipitation assay buffer (Beyotime Institute of Biotechnology). Protein concentration was measured using bicinchoninic acid assay (Beyotime Institute of Biotechnology). Protein samples (30 μg) were loaded onto SDS‐PAGE gel and separated. The samples were subsequently transferred to a PVDF membrane (EMD Millipore). The membranes were then blocked in tris‐buffered saline containing 5% skimmed milk at room temperature for 1 hour, followed by incubation with primary antibodies: RIN1 (1:200; cat. no. ab9485; Abcam Biotechnology Inc.); E‐cad (1:1,000; cat. no. ab15148; Abcam Biotechnology Inc.); Vimentin (1:10000; cat. no. ab24525; Abcam Biotechnology Inc.); Snail (1:300; cat. no. sc‐393172; Santa Cruz Biotechnology Inc.); Bax (1:2000; cat. no. sc‐7480; Abcam Biotechnology Inc.); Cas‐9 (1:5000; cat. no. sc‐73548; Santa Cruz Biotechnology Inc.); MMP9 (1:1000; cat. no. sc‐13520; Santa Cruz Biotechnology Inc.); GAPDH (1:1,000; cat. no. sc‐47724; Santa Cruz Biotechnology Inc.) at 4°C overnight. The following day, the membranes were incubated with horseradish peroxidase‐conjugated secondary antibody (1:5000; cat. no. sc‐2371 or sc‐2357; Santa Cruz Biotechnology Inc.) at room temperature for 2 hours. The protein bands were visualized using an enhanced chemiluminescence protein detection kit (Pierce Biotechnology; Thermo Fisher Scientific, Inc), and the signal was quantified using Image J (NIH).

### 2.8 Assessment of cell viability

The cells were harvested 24 hours post‐transfection. The density of cells seeded onto a 96‐well plate was 3 × 10^4^/well. Cell viability was determined using CCK‐8 assay (Dojindo Molecular Technologies, Inc.) at day 1, 2, 3, and 4 after inoculation. Briefly, 10 µL of CCK‐8 solution was added in each well at different time points. After the incubation at 37°C for another 2 hours, the absorbance (450 nm) was measured by a microplate reader (Bio‐Rad Laboratories, Inc.).

### 2.9 Cell migration assay

The migratory ability of cells was evaluated using wound healing assay. Cells were seeded onto six‐well plates with a density at 2 × 10^5^ cells per well, and subsequently transfected with corresponding vectors. After the cell confluency reached ~90%, the monolayer of cell was scratched with a straight line using a sterile micropipette tip. Then the cells were washed three times with PBS and replenished with fresh culture medium. Later on, the changes of scratch width were monitored at 6, 12, and 24 hours following the scratch. The images were acquired by an inverted microscope (magnification × 100, Olympus Corporation). Cell migration was determined using ImageJ 6.0 with the following formula:

Migration area ratio=the proportion of closed wound area/the whole field of view area

### 2.10 Cell invasion assay

The invasive activity of cells was examined using Transwell assay. A total of 2 × 10^5^ cells were suspended in FBS‐free medium and placed onto the Matrigel^®^‐pre‐coated (Sigma‐Aldrich) upper chamber (BD Biosciences). Then, 500 µL of culture medium supplemented with 10% FBS was added into the lower counterpart. After overnight incubation, non‐invasive cells were removed by a cotton swab, whereas cells invaded into the lower chamber were fixed by paraformaldehyde (4%) and subsequently stained using crystal violet (0.5%). The numbers of invaded cells were counted in five randomly selected fields using an inverted light microscope (magnification×200, Olympus Corporation).

### 2.11 Analysis of cell cycle distribution and apoptosis

Cells treated with o/e‐102209 or o/e‐NC were seeded onto six‐well plates with a density at 2 × 10^5^ cells/well. Later on, cell suspension was spun down using low‐speed centrifugation (1000 rpm) at 4°C for 5 minutees. Cell pellets were rinsed and re‐suspended using PBS, subsequently fixed with pre‐chilled ethanol (70%) and kept in the cold room (4°C) for 2 days. Before being subjected to flow cytometry, cells were lysed, centrifuged, and then re‐suspended in propidium iodide (PI, Sigma‐Aldrich) staining buffer containing PI (50 µL/mL) and RNase A (250 µL/mL). Cell cycle distribution was examined using a flow cytometer (BD Biosciences), and then the results were analysed by Flowjo version 7.6 software (Flowjo LLC). To assess the apoptotic rate, cell suspension was incubated in dark at 4°C for 30 minutes and stained with 5 µL annexin V‐FITC (JingMei Biotech). Cell apoptosis was examined by a flow cytometer (BD Biosciences) and then analysed using Flowjo version 7.6 software (Flowjo LLC).

### 2.12 Animal model

Female BALB/C nude mice (4‐5 weeks old) with the weight of 18‐20 g were obtained from the Laboratory Animal Research Centre of Jinzhou Medical University (Jinzhou, China). The mice were housed under a temperature‐ (22 ± 2°C) and humidity‐controlled (60%) atmosphere, with a 12 hours dark/light cycle and libitum access to food/water for at least 3 days before the operation. Mice were randomly divided into two groups (n = 5/group) and injected with SW48 cells transduced with sh‐NC or sh‐102209. Briefly, 2 × 10^7^ cells were well suspended in 200 μL PBS and then injected into the back of mice subcutaneously. Mice that developed tumors were closely monitored at least four times per week. Six weeks following injection, the mice were sacrificed and tumor tissues were isolated. The volume of tumor was calculated using the formula: V (mm^3^) = (length × width^2^)/2. To induce the metastasis, 1 × 10^5^ cells were suspended using 20 μL PBS and then injected into the lateral tail vein of mice. After inoculation, mice were randomly sorted into experimental groups and assessed 42 days later. Five nude mice were used in each experimental group. The protocol of this study was approved by Ethics Committee of the First Affiliated Hospital of Jinzhou Medical University.

### 2.13 Bioinformatic prediction

Targetscan (www.targetscan.org/) and miRanda (www.microrna.org/microrna/) were used to predict the putative targets of hsa_circRNA_102209 or miR‐761. For luciferase reporter assay, WT fragment of the 3′UTR of hsa_circRNA_102209/RIN1 with potential complementary sites of miR‐761 were purchased from Shanghai GenePharma Co., Ltd. They were integrated into pmirGLO Dual‐Luciferase miRNA Target Expression Vector (Promega Corporation) according to the manufacturer's protocols. Hsa_circRNA_102209/RIN1‐3'UTR‐MUT reporter plasmid that carried the mutant miR‐761 binding site was also produced using QuikChange Multi Site‐Directed Mutagenesis Kit (Stratagene). Subsequently, the corresponding vectors were used to co‐transfect CRC cells with miR‐NC or miR‐761 mimics. Then, luciferase activity was examined 48 hours post‐transfection using Dual Luciferase Reporter Assay System (Promega) according to the manufacturer's protocols, and firefly luciferase activity was normalized to *Renilla* luciferase.

### 2.14 RNA pull‐down assay

Biotinylated miR‐761‐WT, miR‐761‐Mut together with the negative control (GenePharma, Shanghai, China) were produced. Cells were harvested and lysed after 48 h. Cell lysates were indicated with Dynabeads M‐280 Streptavidin. The beads with immobilized miR‐761 were treated using 10 mM ethylenediaminetetraacetic acid. TRIzol^®^ was used to extract the bound RNAs, which were further subjected to RT‐qPCR.

### 2.15 Statistical analysis

Data were presented as means ± standard error of the mean and interpreted using SPSS 17.0 (SPSS, Inc.). The significance of differences within groups was analyzced using Student's t‐test or one‐way analysis of variance (ANOVA). Moreover a student‐Newman‐Keuls test was carried out after ANOVA. The relationship between relative RNA levels was examined by Pearson's correlation analysis. Overall survival was examined using Kaplan‐Meier survival test, and log rank test was used to compare the survival. Chi‐squared test was used to evaluate the relationships among categorical variables. All the experiments were performed in triplicate. *P* < .05 was considered to indicate a statistically significant difference.

## 3 RESULTS

**Figure 1:**
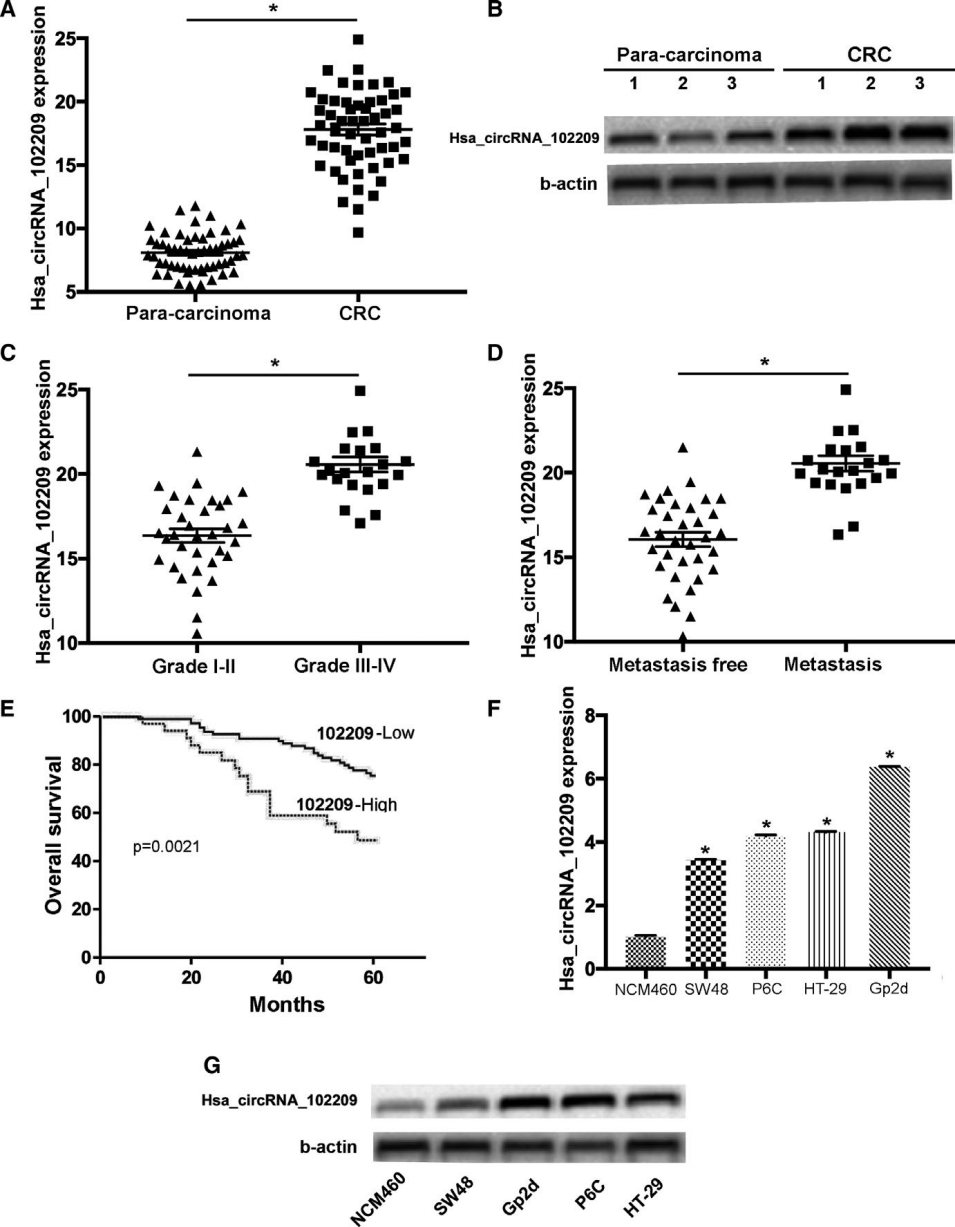
Levels of hsa_circRNA_102209 are upregulated in CRC tissues and cells. (A) The expression of hsa_circRNA_102209 was examined in 56 CRC samples and matched para‐carcinoma tissues by reverse transcription‐quantitative polymerase chain reaction. (B) Northern blotting revealed upregulation of hsa_circRNA_102209 in CRC tissues. (C) Hsa_circRNA_102209 expression was determined in CRC patients with various tumor grading. (D) The levels of hsa_circRNA_102209 were assessed in CRC patients with metastasis compared to the control. (E) Survival analyses of CRC samples with relatively low‐/high‐hsa_circRNA_102209 expression. (F) The expression of hsa_circRNA_102209 was examined in CRC cell lines compared to normal human colonic epithelial cells. (G) Northern blot analysis revealed upregulation of hsa_circRNA_102209 in CRC cells. ^*^ *P* < .05 vs corresponding control. CRC, colorectal cancer

*The level of hsa_circRNA_102209 is elevated in CRC samples and cells*. The expression levels of hsa_circRNA_102209 were examined in 56 CRC samples and matched para‐carcinoma tissues by RT‐qPCR. Our data revealed that hsa_circRNA_102209 was significantly upregulated in CRC tissues compared with para‐carcinoma controls (Figure 1A). In consistence with these results, Northern blotting also revealed upregulation of hsa_circRNA_102209 in CRC specimens (Figure 1B). Furthermore, the association between hsa_circRNA_102209 expression and the progression of CRC was investigated, and the results suggested that the levels of hsa_circRNA_102209 were remarkably increased in patients with advanced CRC (Figure 1C). In addition, the levels of hsa_circRNA_102209 were notably elevated in CRC individuals with liver metastasis (Figure 1D). Moreover CRC patients with high expression levels of hsa_circRNA_102209 exhibited poor overall survival (*P* = .0025, log‐rank test; Figure 1E) compared to the low expression group. Additionally, upregulation of hsa_circRNA_102209 was also detected in CRC cells in comparison with normal colonic epithelial cells (Figure 1E,F). In summary, the expression of hsa_circRNA_102209 was significantly upregulated in CRC, which may also result in tumor progression.

### 3.1 Knockdown of hsa_circRNA_102209 suppresses the growth, metastasis and EMT of CRC cells

**Figure 2:**
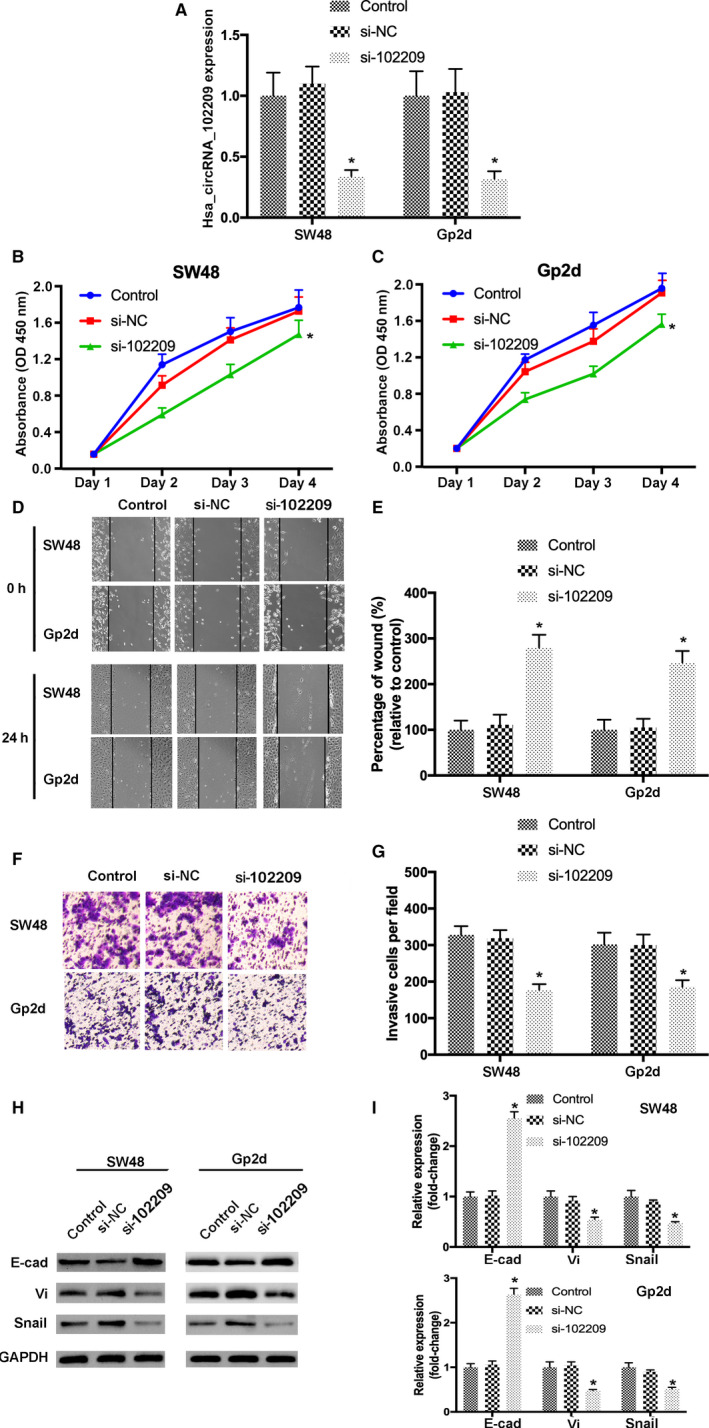
Knockdown of hsa_circRNA_102209 inhibits the proliferation, migration, invasion and EMT of CRC cells. (A) Transfection efficiency of si‐hsa_circRNA_102209 was evaluated by reverse transcription‐quantitative polymerase chain reaction. (B, C) The proliferative activities of CRC cells transfected with si‐hsa_circRNA_102209 or si‐NC were examined using Cell Counting Kit‐8 assay. (D, E) The migratory abilities of transfected SW48 and Gp2d cells were determined by wound healing assay (magnification× 100). (F, G) The invasion of CRC cells transfected with si‐hsa_circRNA_102209 or si‐NC were evaluated (magnificationx200). (H, I) The levels of EMT‐related molecules were assessed using both RT‐qPCR and western blotting. ^*^ *P* < .05 vs nontransfected cells. CRC, colorectal cancer; NC, negative control

To study the influences of hsa_circRNA_102209 on the biological behaviors of CRC cells, the expression of hsa_circRNA_102209 was knockdown in SW48 and Gp2d cells. Transfection efficiencies were determined by RT‐qPCR (Figure 2A). In addition, the data of CCK‐8 assay indicated that the proliferative activity of CRC cells transfected with si‐hsa_circRNA_102209 was remarkably inhibited (Figure 2B,C). Furthermore, would healing and Transwell assays were carried out to evaluate cell migration and invasion, respectively. The results revealed that cell migration and invasion were inhibited following the knockdown of hsa_circRNA_102209 compared to the control (Figure 2D‐G). Additionally, in order to investigate the effects of hsa_circRNA_102209 knockdown on EMT of CRC cells, the expression levels of relevant markers including E‐cad, vimentin and snail were examined. The protein and mRNA levels of abovementioned molecules were affected after the transfection with si‐hsa_circRNA_102209 (Figure 2H,I). Taken all together, knockdown of hsa_circRNA_102209 could lead to downregulated proliferation, migration, invasion and EMT of CRC cells.

### 3.2 Downregulated hsa_circRNA_102209 enhances cell cycle arrest and apoptosis in CRC cells

**Figure 3:**
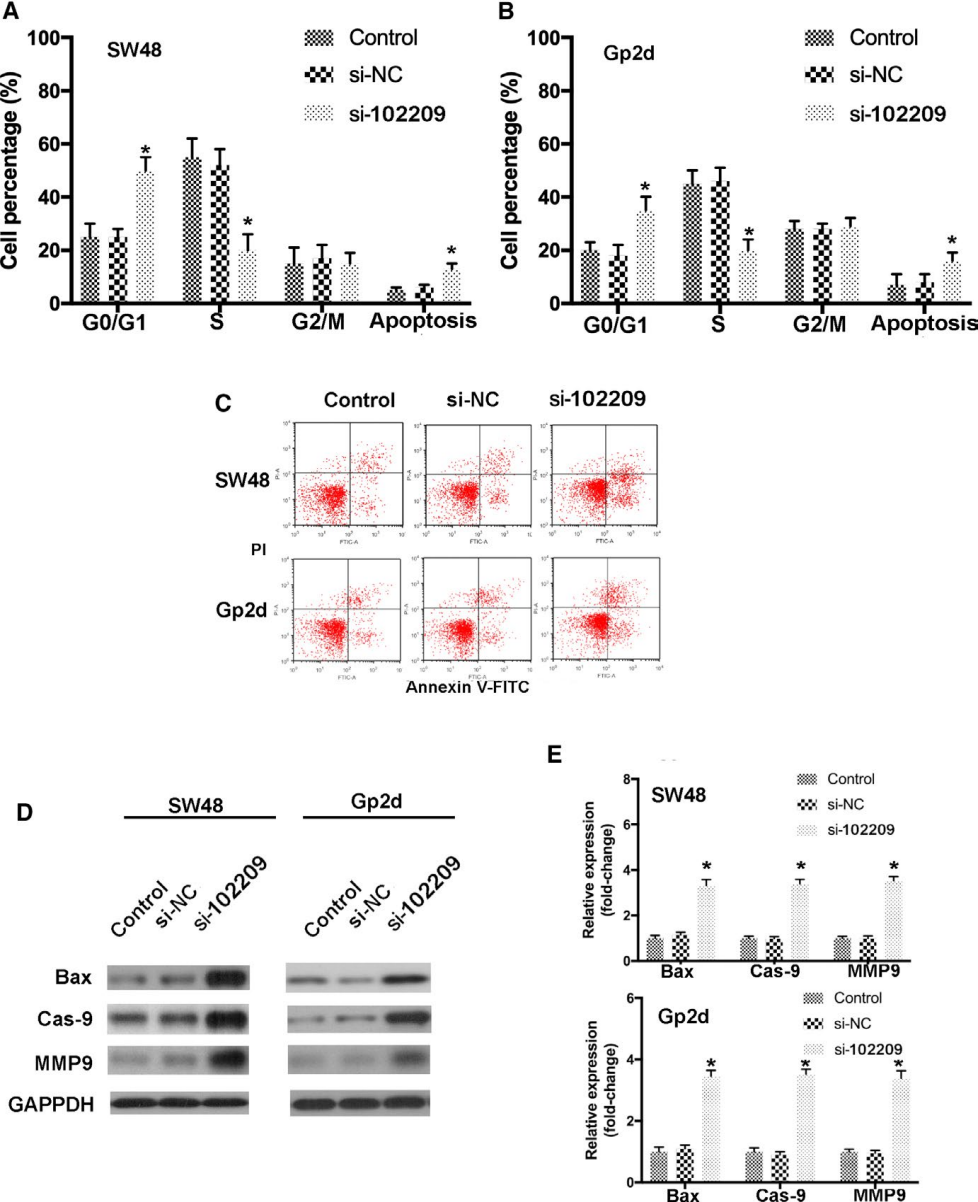
Knockdown of hsa_circRNA_102209 promotes cell cycle arrest and apoptosis in CRC cells. (A, B) The distribution of cell cycle and apoptotic rates of CRC cells with hsa_circRNA_102209 knockdown were evaluated. (C) The apoptosis of SW48 and Gp2d cells transfected with si‐hsa_circRNA_102209 were also examined by flow cytometry. (D and E) The expression profiles of apoptosis‐related molecules were determined in CRC cells with hsa_circRNA_102209 knockdown compared to the control. ^*^ *P* < 0.05 vs nontransfected cells. CRC, colorectal cancer

Based on the abovementioned findings, hsa_circRNA_102209 may be involved in the proliferation and metastasis of CRC cells in vitro. To further determine the influences of hsa_circRNA_102209 knockdown, cell cycle distribution and apoptosis in transfected CRC cells were also examined compared to the control. Our data suggested that CRC cell cycle was notably shifted from S and G2/M to G0/G1 phase, as the cell proportion of G0/G1 phase was significantly elevated, while that in S phase was remarkably decreased (Figure 3A,B). In addition, the results of flow cytometry indicated that knockdown of hsa_circRNA_102209 increased the apoptotic rate of CRC cells (Figure 3C), which was further confirmed by the upregulation of apoptosis‐associated molecules such as Bax, Cas‐9 and MMP9 (Figure 3D,E). In summary, these data revealed that hsa_circRNA_102209 knockdown may arrest cell cycle at G0/G1 phase, consequently promoting the apoptosis of CRC cells.

### 3.3 MiR‐761 is the novel downstream molecule of hsa_circRNA_102209 in CRC

**Figure 4:**
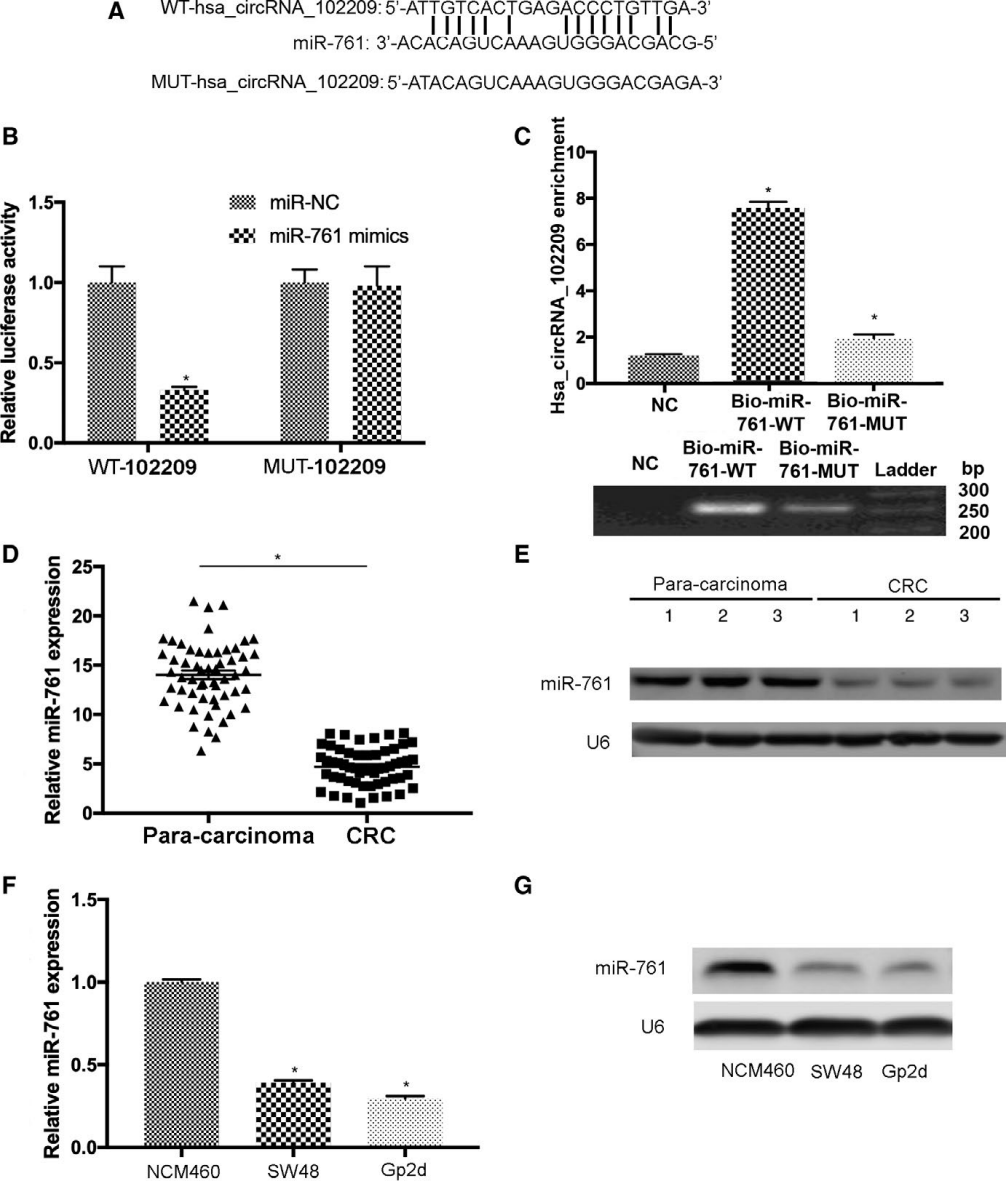
MiR‐761 is the putative target of hsa_circRNA_102209 in CRC cells. (A) The complementary binding sites between hsa_circRNA_102209 and miR‐761 were predicted. (B) The association between hsa_circRNA_102209 and miR‐761 was further confirmed by luciferase reporter assay. (C) In RNA pull‐down assay, hsa_circRNA_102209 was enriched in biotin‐labeled miR‐761‐WT group. (D‐G) The expression levels of miR‐761 were decreased in CRC clinical samples and cell lines. ^*^ *P* < 0.05 vs corresponding control. CRC, colorectal cancer; NC, negative control

To investigate whether hsa_circRNA_102209 is a putative oncogenic factor in CRC and its roles via targeting downstream miRNAs, the potential targets of has_circ_102209 including miR‐761, miR‐197‐3p, miR‐204‐5p, miR‐211‐5p, and miR‐134‐5p were predicted using bioinformatics approaches. Among them, the 3′‐UTR of miR‐761 contained a highly conserved binding site for has_circRNA_102209 (Figure 4A). Moreover the relationship between hsa_circRNA_102209 and miR‐761 was confirmed by luciferase activity assay. The plasmids carrying wild‐type (WT‐hsa_circRNA_102209) and mutant (MUT‐hsa_circRNA_102209) sequence of predicted miR‐761 complementary sites were produced. The results indicated that miR‐761 mimics remarkably decreased the activity of WT‐hsa_circRNA_102209 luciferase reporter by comparing to the control (Figure 4B). RNA pull‐down assay revealed that hsa_circRNA_102209 was enriched in biotin‐labeled miR‐761‐WT group (Figure 4C). Additionally, the data of RT‐qPCR and northern blotting revealed that the expression levels of miR‐761 were downregulated in CRC tissues (Figure 4D,E). Furthermore, downregulation of miR‐761 was observed in SW48 and Gp2d cells (Figure 4F,G).

**Figure 5:**
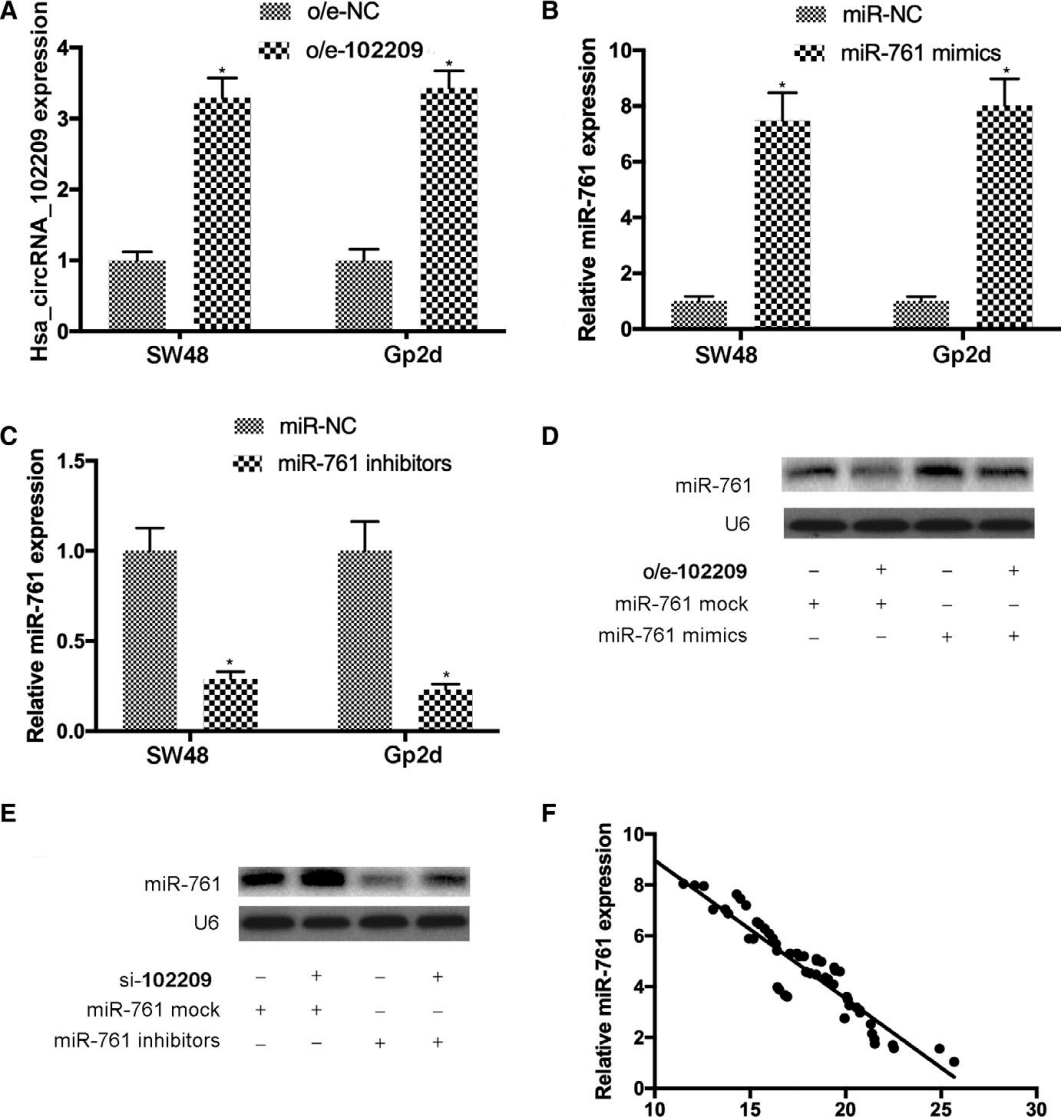
Expression of miR‐761 was downregulated by hsa_circRNA_102209 in CRC. (A‐C) The transfection efficiencies of o/e‐102209, miR‐761 mimics and inhibitors were confirmed by RT‐qPCR. (D and E) The levels of miR‐176 were negatively regulated by hsa_circRNA_102209 in CRC cells. (F) The expression of hsa_circRNA_102209 and miR‐761 were inversely correlated in CRC samples (*r* = −.3223; *P* = .00909). ^*^ *P* < 0.05 vs o/e‐NC or miR‐NC. CRC, colorectal cancer

To further investigate the influences of hsa_circRNA_102209 on the expression of miR‐761, SW48 cells were co‐transfected with o/e‐102209 and miR‐761 mimics, or with si‐hsa_circRNA_102209 and miR‐761 inhibitors, respectively. The transfection efficiencies were confirmed using RT‐qPCR (Figure 5A‐C). The results of northern blot analysis also suggested that the upregulation of miR‐761 in SW48 cells transfected with miR‐761 mimics was abolished by o/e‐102209 (Figure 5D). Vice versa, downregulation of miR‐761 in SW48 cells transfected with miR‐761 inhibitors was reversed after co‐transfection with si‐hsa_circRNA_102209 (Figure 5E). Furthermore, the association between hsa_circRNA_102209 and miR‐761 was further determined by Pearson's correlation analysis, and the results suggested that the expression levels of miR‐761 and hsa_circRNA_102209 were inversely correlated in CRC tissues (Figure 5F).

### 3.4 RIN1 is a potential target of miR‐761 in CRC

**Figure 6:**
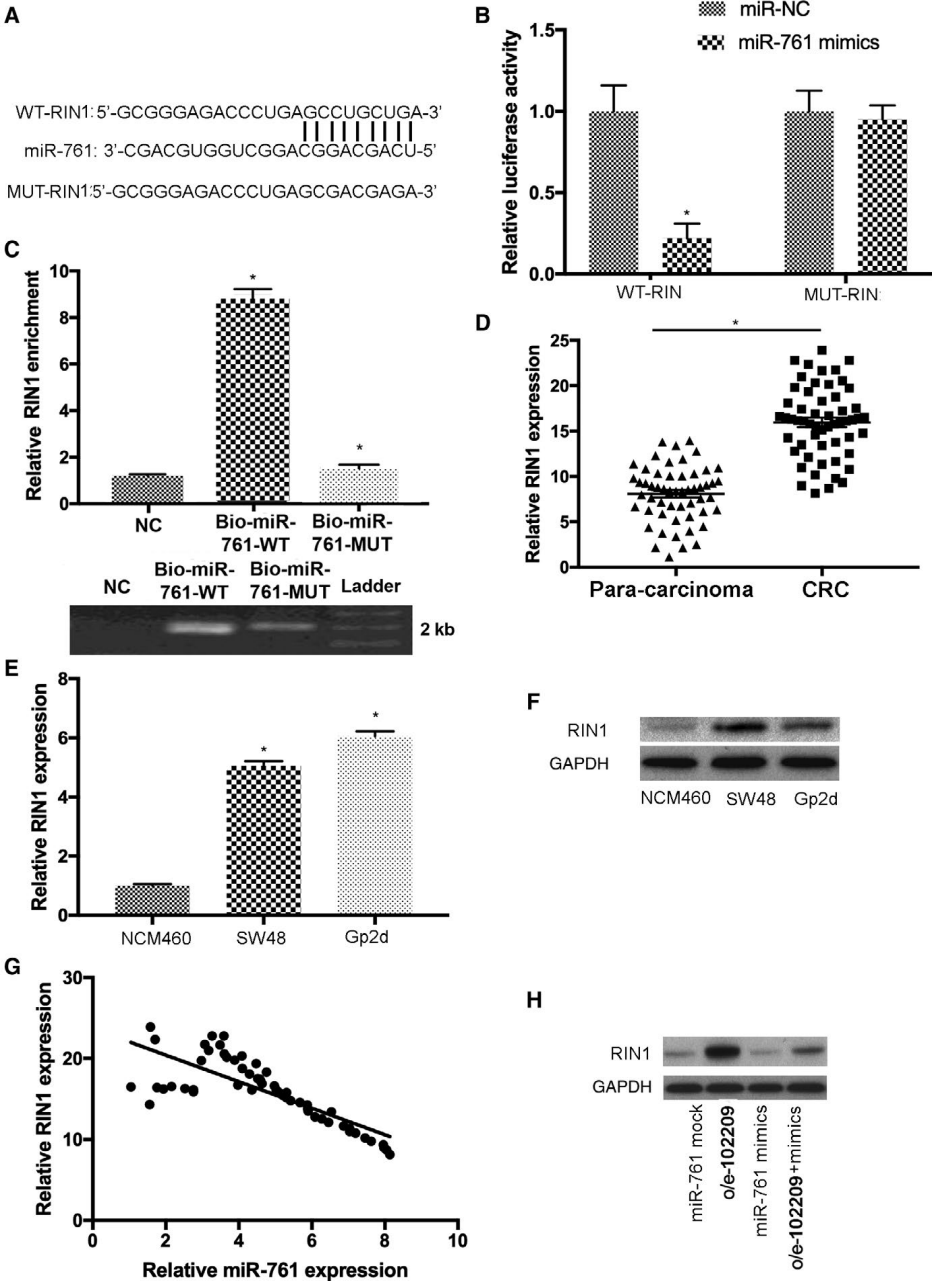
RIN1 is a novel downstream molecule of miR‐761 in CRC cells. A, The binding sites of RIN1 on the transcript of miR‐761 were predicted. B, The correlation between RIN1 and miR‐761 was elucidated using luciferase activity assay. C, In RNA pull‐down assay, hsa_circRNA_102209 was enriched in biotin‐labeled miR‐761‐WT group. (D‐F) RIN1 expression was elevated in both CRC tissues and cells. (G) The levels of RIN1 and miR‐761 were inversely correlated in CRC (*r* = −.3555; *P* = .00912). (H) The levels of RIN1 were upregulated by hsa_circRNA_102209 and downregulated by miR‐761 in CRC cells, respectively. ^*^ *P* < .05 vs corresponding control. CRC, colorectal cancer

To identify the putative downstream molecule of miR‐761, bioinformatics study was performed and several pathways were identified. Among them, miR‐761 is able to inhibit RIN1 signaling and regulate the development of gastric cancer,^26^ thus it is selected for further study. The complementary sequence between miR‐761 and RIN1 transcripts was predicted (Figure 6A). The relationship of RIN1 and miR‐761 was also revealed by luciferase assay. The vectors containing the wild‐type (WT‐RIN1) and mutant (MUT‐RIN1) sequence of predicted miR‐761 binding sites were generated. The results indicated that overexpression of miR‐761 significantly suppressed the activity of luciferase reporters carrying WT‐RIN1 sequence but not in mutant group (Figure 6B). RNA pull‐down assay indicated that RIN1 was enriched in biotin‐labeled miR‐761‐WT group (Figure 6C). In addition, the results of RT‐qPCR suggested that the levels of RIN1 increased in CRC tissues compared to normal controls (Figure 6D). Furthermore, upregulation of RIN1 was observed in both SW48 and Gp2d cells (Figure 6E,F). Moreover, the expression levels between RIN1 and miR‐761 were negatively correlated in CRC samples (Figure 6G). The expression levels of RIN1 were remarkably increased in CRC cells treated with o/e‐102209; significant downregulation of RIN1 was detected in CRC cells following the transfection with miR‐761 mimics, which was restored by overexpression of hsa_circRNA_102209 (Figure 6H). In summary, hsa_circRNA_102209 could increase the expression levels of RIN1 in CRC cells by sponging miR‐761.

### 3.5 RIN1 could be involved in hsa_circRNA_102209‐modulated biological behavior changes in CRC cells

**Figure 7:**
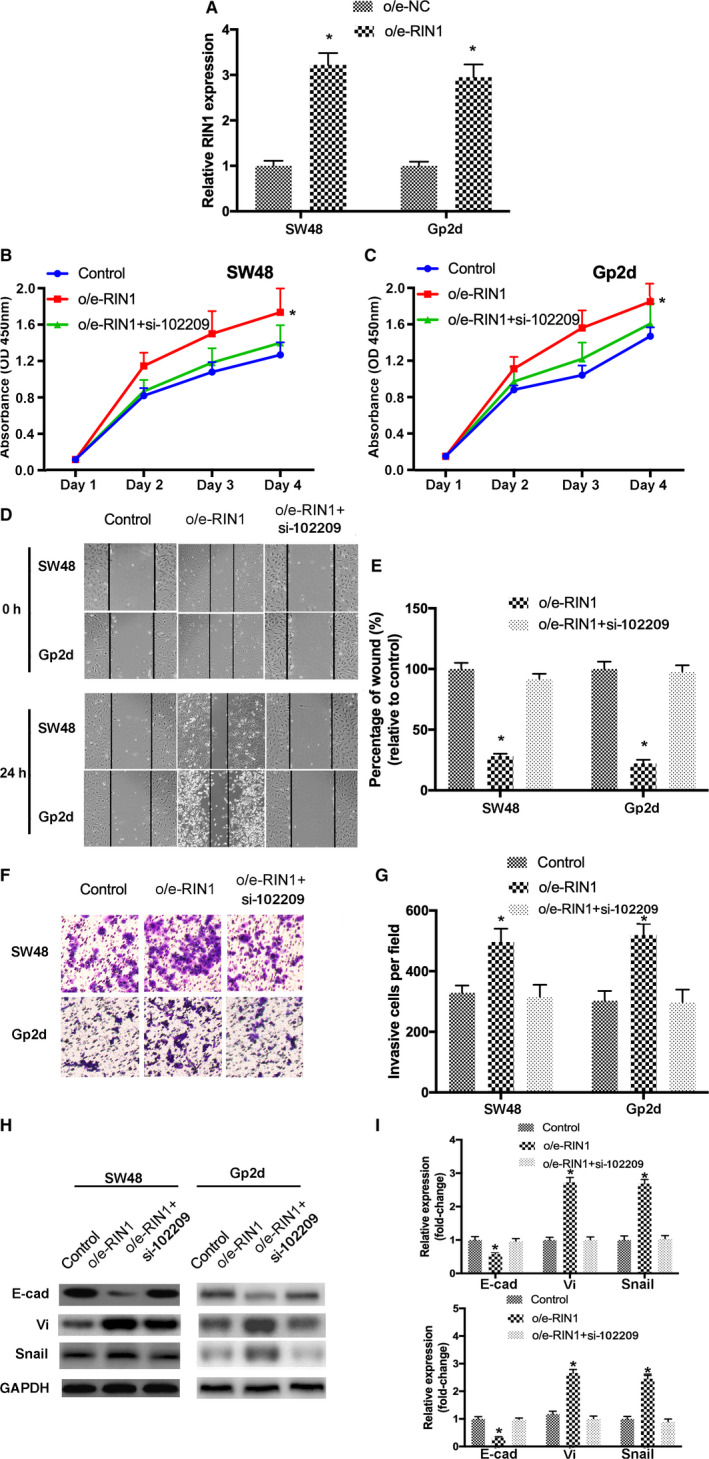
RIN1 could contribute to hsa_circRNA_102209‐induced biological behavior changes in CRC cells. (A) Transfection efficiency of o/e‐RIN1 was confirmed using RT‐qPCR. (B‐G) The proliferative, migratory and invasive activity of CRC cells were enhanced by o/e‐RIN1, which was reversed by si‐hsa_circRNA_102209. (H and I) The levels of EMT‐related molecules were also determined in transfected CRC cells. ^*^ *P* < .05 vs o/e‐NC or nontransfected cells. CRC, colorectal cancer; NC, negative control

To investigate whether hsa_circRNA_102209‐mediated upregulation of RIN1 is associated with the development of CRC, CRC cells were transfected with control vector, o/e‐RIN1, or co‐treated with si‐hsa_circRNA_102209. Transfection efficiencies were determined by RT‐qPCR (Figure 7A). In addition, CCK‐8 assay revealed that the proliferation of CRC cells transfected with o/e‐RIN1 was significantly promoted, which was abrogated by hsa_circRNA_102209 knockdown (Figure 7B,C). Similarly, wound healing and Transwell assays indicated that cell migration and invasion were enhanced by overexpressed RIN1, but these effects were notably reversed by the transfection with si‐hsa_circRNA_102209 (Figure 7D‐G). Furthermore, the expression levels of EMT‐associated molecules were also influenced by overexpression of RIN1, which was abolished after the transfection with si‐hsa_circRNA_102209 (Figure 7H,I).

**Figure 8:**
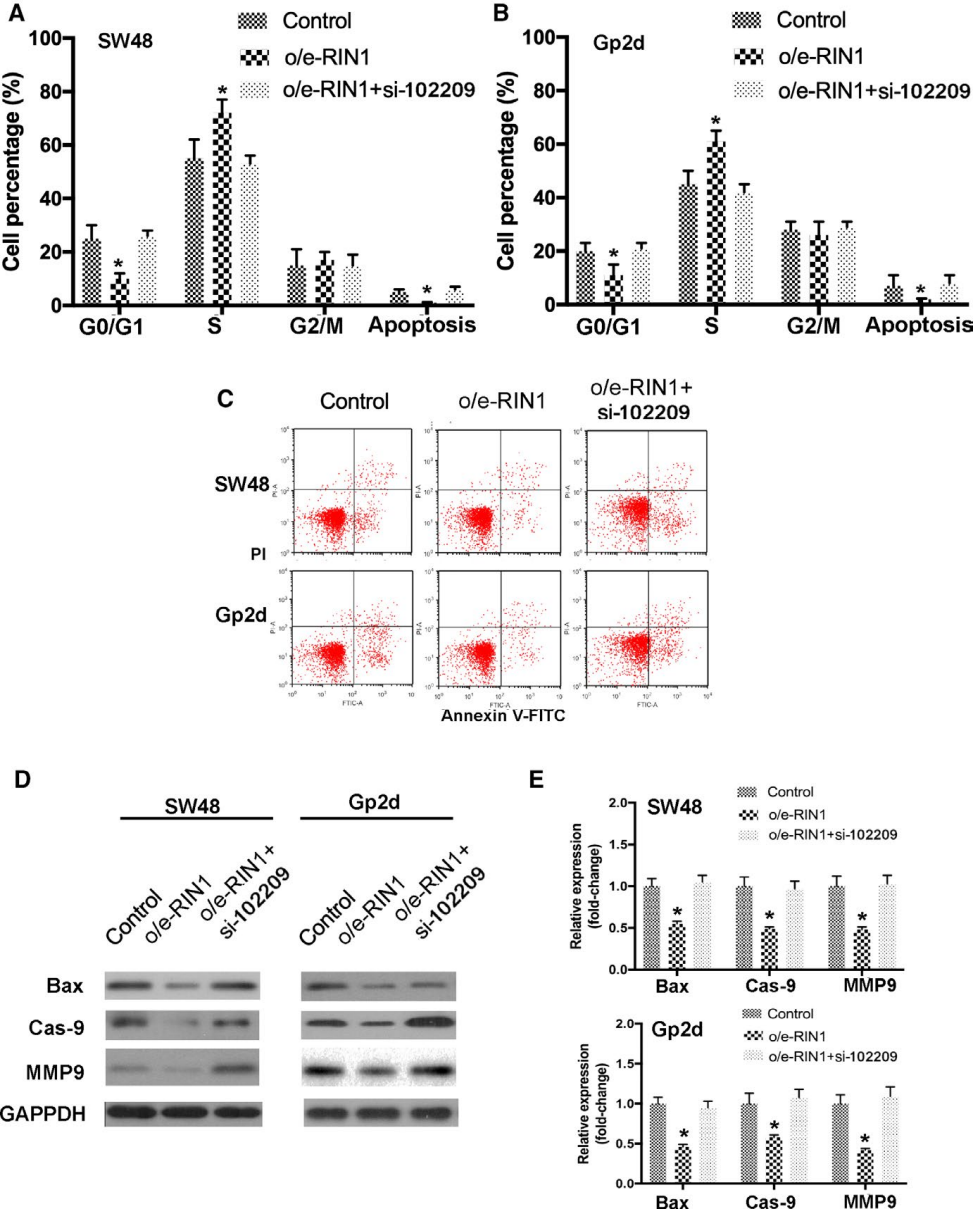
RIN1 is involved in hsa_circRNA_102209‐modulated cell cycle arrest and apoptosis in CRC. (A and B) Overexpression of RIN1 suppressed cell cycle arrest in CRC cells, which was abrogated by hsa_circRNA_102209 knockdown. (C‐E) The apoptosis of CRC cells was inhibited by the transfection with o/e‐RIN1, and these effects were abolished by knockdown of hsa_circRNA_102209. ^*^ *P* < .05 vs nontransfected cells. CRC, colorectal cancer

Moreover cycle distribution and apoptosis in transfected CRC cells were also determined. Our data revealed that cell cycle was shifted from G0/G1 to S and G2/M phase in CRC cells transfected with o/e‐RIN1, which were reversed by knockdown of hsa_circRNA_102209 expression. In addition, the cell percentage of G0/G1 phase was remarkably reduced by o/e‐RIN1, but these effects were abrogated by knockdown of hsa_circRNA_102209 (Figure 8A,B). In addition, the results of flow cytometry suggested that overexpression of RIN1 inhibited the apoptosis of CRC cells, which was abolished by the transfection with si‐hsa_circRNA_102209 (Figure 8C). These findings were further confirmed by the changes of apoptosis‐related markers including Bax, Cas‐9. and MMP9 (Figure 8D,E). Taken all together, the biological behavior changes caused by overexpressed RIN1 were abrogated by knockdown of hsa_circRNA_102209. These findings revealed that RIN1 is involved in hsa_circRNA_102209‐mediated growth and metastasis of CRC cells, suggesting hsa_circRNA_102209/miR‐761/RIN1 axis could contribute to the progression of CRC.

### 3.6 Knockdown of hsa_circRNA_102209 suppresses the development of CRC in vivo

**Figure 9:**
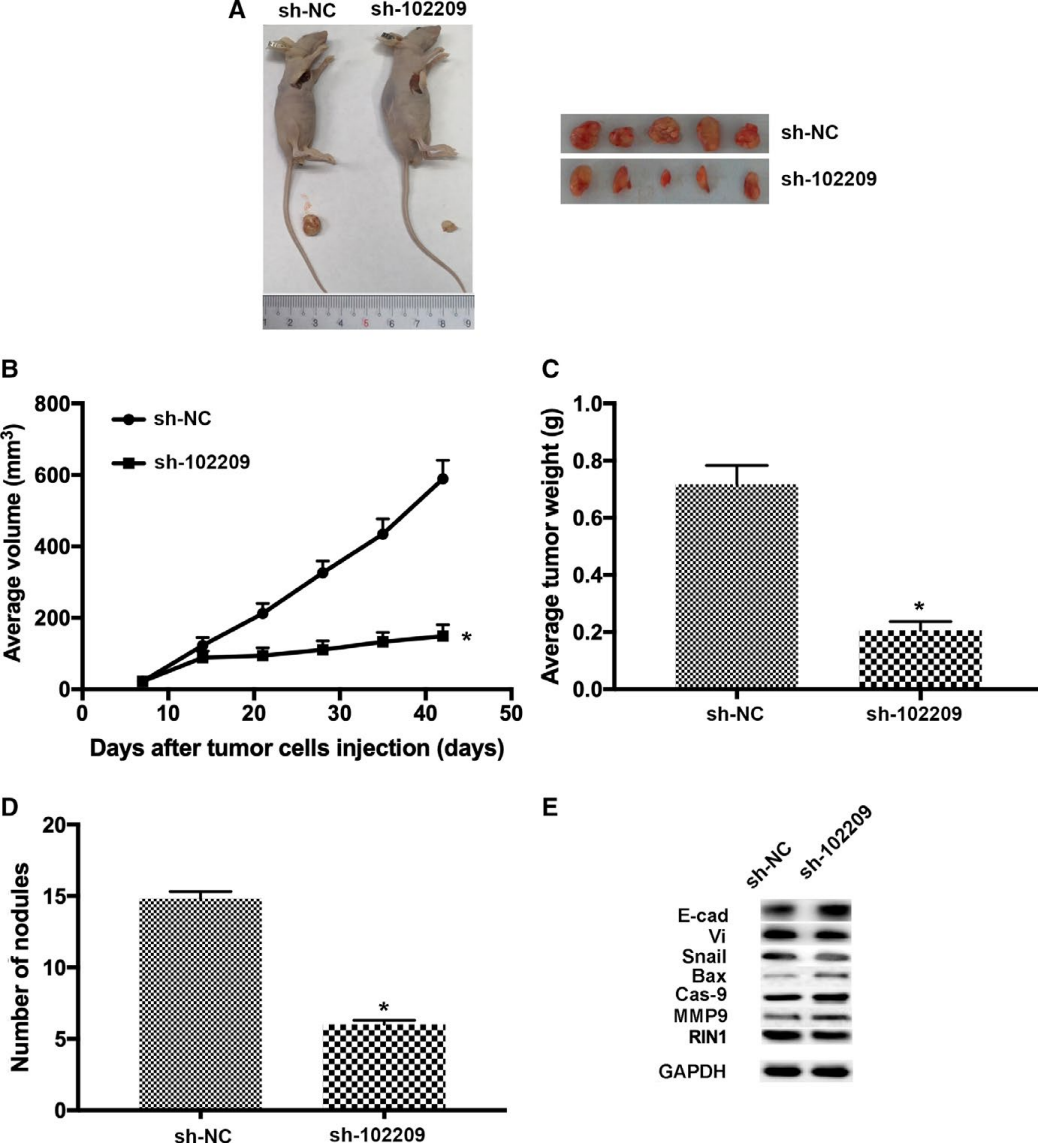
Knockdown of hsa_circRNA_102209 suppressed the development of CRC in mouse model. (A) Six weeks post‐injection, the mice were sacrificed and tumor tissues were examined. (B and C) The average tumor weight and volume were both significantly reduced in hsa_circRNA_102209 knockdown mice. (D) The numbers of macroscopic nodules in hsa_circRNA_102209 knockdown group was also examined compared to the control. (E) The levels of EMT‐/apoptosis‐markers and RIN1 exhibited the similar expression pattern as observed in the in vitro assays. ^*^ *P* < .05 vs si‐NC. CRC, colorectal cancer; NC, negative control

To study whether si‐102209 is able to inhibit the growth and metastasis of CRC in vivo, cells transfected with si‐NC or si‐102209 were subcutaneously injected into BALB/C nude mice. Six weeks post‐inoculation, the mice were sacrificed and tumor tissues were isolated and further examined (Figure 9A). The mean value of tumor volume in si‐102209 group was remarkably reduced compared to the control group (Figure 9B). In addition, the average tumor weight of hsa_circRNA_102209 knockdown mice were notably decreased (Figure 9C). Furthermore, the numbers of macroscopic nodules were significantly reduced in si‐102209 group (Figure 9D). The results of western blotting also indicated that EMT‐/apoptosis‐related markers and RIN1 exhibited similar expression pattern as detected in the in vitro assays (Figure 9E). Taken all together, our findings indicated that knockdown of hsa_circRNA_102209 was able to inhibit the progression of CRC in vivo possibly through downregulating RIN1.

## 4 DISCUSSION

CRC is the third most common malignancy worldwide, and ~1.4 million new cases are diagnosed globally every year.^1^ Recent research suggested that genetic/epigenetic alterations could trigger the progression of CRC.^2^, ^3^, ^4^ Moreover the survival rate for CRC patients with metastases remains poor (<10%; 9). The detailed mechanisms underlying the pathogenesis of CRC are still unknown, therefore, it is urgent to discover novel diagnostic biomarkers for this disease. Recently, accumulating evidences have revealed that circRNAs could be key regulators during the progression of cancer. They may function as putative tumor promoters/suppressors, and impaired expression levels of circRNAs could lead to tumorigenesis.^10^, ^11^, ^12^, ^13^ Additionally, circRNAs are able to function as “sponges’ of certain miRNAs, consequently inhibiting their activities.^14^ For example, circRNAs GLI2 could promote the growth of osteosarcoma by modulating miR‐125b‐5p.^17^ Furthermore, circRNA ZKSCAN1 is capable of suppressing the growth and metastasis of hepatocellular carcinoma cells.^16^ However, the underlying mechanisms and novel targets of circRNAs in CRC are not completely understood. Previous studies have suggested that miRNAs were associated with the pathogenesis of cancer.^20^, ^21^, ^22^, ^23^, ^24^, ^25^ MiRNAs are promising oncogenic factors or tumor suppressors; they function as essential regulators of gene expression and are putative targets of circRNAs.^19^, ^20^, ^21^, ^22^, ^23^, ^24^ For instance, miR‐93 is able to enhance the proliferation of glioma cells by targeting phosphatidylinositol 3 kinase/protein kinase B pathway.^22^ In addition, miR‐140 and miR‐152 could interact with certain lncRNAs during tumorigenesis^24^, ^25^; however, the exact functions of miRNAs in CRC remain largely unknown and require further investigation. Among the above‐mentioned miRNAs, miR‐761 is associated with the progression of gastric cancer by suppressing RIN1.^26^

In our paper, increased expression levels of hsa_circRNA_102209 were observed in CRC samples and cell lines. Knockdown of hsa_circRNA_102209 inhibited the growth and metastasis of CRC cells in vitro. In addition, function studies were conducted to investigate the downstream molecules and signaling of hsa_circRNA_102209 in CRC. Our data suggested that hsa_circRNA_102209 binds to miR‐761, whose levels were notably reduced in CRC tissues and cells. Furthermore, the levels of miR‐761 were downregulated by hsa_circRNA_102209 in CRC cells, and their expression were negatively correlated in CRC samples. Similarly, involvement of other circRNAs has also been reported in the development of CRC. For example, novel circRNA CDR1as is able to suppress the progression of CRC through downregulating miR‐7 and upregulating EGFR/IGF‐1R, respectively.^27^ Moreover circRNAs including hsa_circ_0000523, circ_ITCH and hsa_circ_0007142 are involved in the pathogenesis of CRC by regulating tumor cell growth.^28^, ^29^, ^30^ Hsa_circ_0007142 was able to promote the development of CRC via suppressing miR‐103A‐2‐5p.^28^ Circ_ITCH exhibited inhibitory roles in CRC by regulating Wnt/b‐catenin signalling.^29^ Has_circ_0000523 was a novel sponge of miR‐31, and downregulated hsa_circ_0000523 resulted in activation of Wnt/b‐catenin pathway, consequently promoting the development of CRC. Furthermore, previous study has also revealed that hsa_circ_0007534 is associated with the initiation and development of CRC by targeting Bcl‐2, as silenced hsa_circ_0007534 inhibited the proliferation and promote the apoptosis of CRC cells.^18^

Additionally, RIN1 was identified as the putative target of miR‐761, and its expression levels were notably increased in CRC samples and cells. The levels of RIN1 were upregulated by hsa_circRNA_102209 and downregulated by miR‐761 in CRC cells, respectively. Furthermore, the growth and development of CRC cells could be enhanced by overexpressed RIN1, and these effects were remarkably abrogated by hsa_circRNA_102209 knockdown. These data indicated that RIN1 could be involved in tumor cell growth and metastasis during the progression of CRC. In consistence with our findings, previous study has reported that miR‐761 is associated with the development of gastric cancer by regulating RIN1.^26^

Our data revealed that hsa_circRNA_102209 was a novel oncogenic factor in CRC that could promote the growth and metastasis of tumor cells by suppressing miR‐761 and upregulating RIN1. The above‐mentioned findings revealed the key roles of hsa_circRNA_102209 on tumorigenesis and elucidated the potential mechanisms underlying its regulatory functions. However, there are some limitations in the present study, for instance, future work may be required to investigate whether the expression profiles of hsa_circRNA_102209, miR‐761 and RIN1 are differentiated between CRC cases with metastasis to other organs and non‐metastatic group, if so, this novel signaling could also be with therapeutic usefulness for the treatment of metastatic cases to other organs. Additionally, in order to confirm the existing findings of CCK‐8 and Transwell assay, the expression levels of proliferation‐ and invasion‐associated molecules could be evaluated in transfected cells, respectively. Taken all together, our study indicated that hsa_circRNA_102209/miR‐761/RIN1 axis could be associated with the progression of CRC. More importantly, and this novel signaling pathway could be a promising therapeutic target for the treatment of patients with CRC. For instance, miRNA‐targeted therapy such as treatment with synthetic miR‐761 mimics could be considered in future.

## CONFLICT OF INTEREST

The authors declare that they have no competing interests.

## AUTHORS’ CONTRIBUTION

CL and HZ designed this study. Both authors carried out the experiments and analyzed the data. CL and HZ drafted the original manuscript, reviewed, and approved the final version of this manuscript.

## ETHICAL APPROVAL

The protocol was approved by the Ethics Committee of the First Affiliated Hospital of Jinzhou Medical University (Jinzhou, China). Written informed consents were received from all patients prior to sample collection.

## Data Availability

All the original datasets generated or analyzed in our study are available upon reasonable request.
